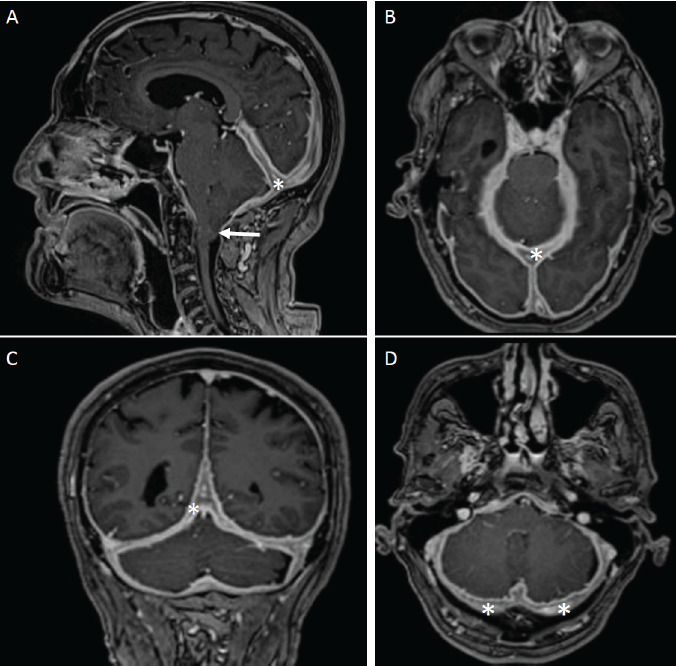# Letter to the Editor: Idiopathic Hypertrophic Pachymeningitis presenting as Occipital Neuralgia with associated Chiari Malformation

**DOI:** 10.4314/ahs.v24i2.16

**Published:** 2024-06

**Authors:** José L Ruiz-Sandoval, Marco A Sánchez Torres, Jefte Felipe Uribe-Martínez, Amado Jimenez-Ruiz

**Affiliations:** 1 Servicio de Neurología, Hospital Civil de Guadalajara “Fray Antonio Alcalde”. Guadalajara, Jalisco, Mexico; 2 Departamento de Neurociencias, Centro Universitario de Ciencias de la Salud, Universidad de Guadalajara, Guadalajara, Jalisco, México; 3 Servicio de Reumatología, Hospital Civil de Guadalajara “Fray Antonio Alcalde”. Guadalajara, Jalisco, México

**Keywords:** Pachymeningitis, headache, Neuralgia

We read the case report published by Auboire et al., regarding a case of idiopathic pachymeningitis, presenting as occipital neuralgia^1^. We would like to enrich the presented discussion by presenting a recent case seen in our Neurology Department, with similar clinical features, but distinct radiologic findings.

A previously healthy 76-year-old man, with a history of a three-year progressively worsening headache, was seen in the out-patient neurology clinic. Headache semiology included short (a few seconds) bouts of paroxistic occipital “stabbing” pain, related to positional head movements. The pain radiated to the superficial posterior and anterior part of the right scalp, with an accompanying cramplike sensation in the right occiput, a presentation consistent with occipital neuralgia (Arold's Neuralgia). He also experienced vertigo, dysarthria, and right arm incoordination. A contrast-enhanced cranial MRI showed diffuse regular pachymeningeal enhancement (Panel A, B, C, and D; asterisk), and a 12-mm caudal cerebellar tonsil descent (type 1 Arnold-Chiari malformation) (Panel A, Arrow). Immunological workup was normal (including IgG-4 quantification), and other infectious and neoplastic causes were reasonably excluded (in a resource-limited setting). A meningeal biopsy was unavailable, due to the ongoing COVID-19 pandemic. We established a diagnosis of idiopathic hypertrophic cranial pachymeningitis (IHCP). After receiving 50mg prednisone per day for three months, the patient experienced complete remission of the observed symptoms, and was lost to follow-up.

IHCP is an inflammatory condition, characterized by localized or diffuse thickening of the dura mater in the brain or upper spinal cord regions^2^. It may be secondary to inflammatory, infectious, or neoplastic causes. When none are found, it may be classified as a primary disorder^3^. It usually presents with headache and cranial nerve palsies, but can involve posterior fossa structures, and present clinically with cerebellar dysfunction. Occipital neuralgia is a distinctive form of headache, involving the posterior head in the distributions of the occipital nerves (greater occipital, lesser occipital, and third occipital). A secondary cause of occipital nerve damage must always be considered, such as the one presented here. IHCP affects older patients, and usually has an excellent response to steroids and immunosuppression. To our knowledge, this is the first case description of IHCP presenting with occipital neuralgia and associated cerebellar descent.

**Figure:**